# Insights into Differentiation of Melanocytes from Human Stem Cells and Their Relevance for Melanoma Treatment

**DOI:** 10.3390/cancers12092508

**Published:** 2020-09-03

**Authors:** Madalina A. Mirea, Stefan Eckensperger, Markus Hengstschläger, Mario Mikula

**Affiliations:** Institute for Medical Genetics, Center for Pathobiochemistry and Genetics, Medical University Vienna, Währingerstrasse 10, 1090 Vienna, Austria; madalina.mirea@meduniwien.ac.at (M.A.M.); stefan.eckensperger@gmx.at (S.E.); markus.hengstschlaeger@meduniwien.ac.at (M.H.)

**Keywords:** melanocyte differentiation, pluripotent embryonic stem cells, EMT, melanoma metastasis

## Abstract

**Simple Summary:**

The reactivation of embryonic developmental programs is crucial for melanoma cells to grow and to metastasize. In order to understand this process better, we first summarize the melanocytic differentiation process both *in vivo* and *in vitro*. Secondly, we compare and highlight important similarities between neural crest cell fate during differentiation and tumor cell characteristics during melanoma mestastasis. Finally, we suggest possible therapeutic targets, which could be used to inhibit phenotype switching by developmental cues and hence also suppress the metastatic melanoma spread.

**Abstract:**

Malignant melanoma represents a highly aggressive form of skin cancer. The metastatic process itself is mostly governed by the so-called epithelial mesenchymal transition (EMT), which confers cancer cells migrative, invasive and resistance abilities. Since EMT represents a conserved developmental process, it is worthwhile further examining the nature of early developmental steps fundamental for melanocyte differentiation. This can be done either *in vivo* by analyzing the physiologic embryo development in different species or by *in vitro* studies of melanocytic differentiation originating from embryonic human stem cells. Most importantly, external cues drive progenitor cell differentiation, which can be divided in stages favoring neural crest specification or melanocytic differentiation and proliferation. In this review, we describe ectopic factors which drive human pluripotent stem cell differentiation to melanocytes in 2D, as well as in organoid models. Furthermore, we compare developmental mechanisms with processes described to occur during melanoma development. Finally, we suggest differentiation factors as potential co-treatment options for metastatic melanoma patients.

## 1. Introduction

Melanoma arises from melanocytes and represents the most dangerous form of skin cancer, as it frequently metastasizes to distant organs. Melanoma is generally hallmarked by activating MAPK (Mitogen Activated Protein Kinase) mutations and loss of the cell cycle suppressor gene CDKN2A, while functional TP53 is often retained [1,2]. A recent study analyzed the mutation profile in 331 patients and found melanoma subtypes based on mutation patterns, with 52% of cases hallmarked by a V600E mutation in BRAF, 28% showing mutations in NRAS and 14% in NF1 [3].

The metastatic process occurring during melanoma progression has been described as a switch in the tumor phenotype from a mainly proliferating type to a highly invasive type [4]. Importantly, this process is characterized by onset and reversion of epithelial mesenchymal transition (EMT) in a repeating process. Hence, phenotype switching can occur multiple times at different sites in the human body. It is postulated that this process is one of the main reasons for the aggressiveness of human malignant melanoma and it has been shown that the transcription factors TWIST, ZEB1 and STAT3 are its main drivers [5,6,7].

During development, EMT enables epithelial cells of different tissues to become migratory and have the capacity to move freely through the extracellular matrix [8,9]. This process can be fully reversed by counter regulation of the same genes [10,11]. During EMT, cells lose their cell-cell-junctions [12], as TGFβ recruits TGFBR2, which phosphorylates PAR6 and which then leads to the degradation of RhoA [13], an essential protein for these junctions. Cells also lose their adherence to the cellular matrix [12] by internalization of E- and β-catenin [14,15] because of the induction by growth factors and subsequent internalization and degradation [16].

EMT itself is a well described sequence of events in which cell adhesion molecules like E-cadherin, claudins, occludin, plakophilin and desmoplakin become down-regulated, thus destabilizing cell-cell adhesion and cell junctions [17,18,19]. In this process, the Snail family of zinc-finger transcription factors and Twist, a basic helix-loop-helix transcription factor, have an essential role, converting epithelial cells into migratory mesenchymal cells [20,21]. These factors are not only important for organogenesis during embryonic development and wound healing later on, but can also be used by tumor cells to acquire invasive properties. The main function of Snail and Twist is to down-regulate E-cadherin expression and up-regulate mesenchymal proteins including N-cadherin. This changes the adhesive properties of the cell in a process referred to as cadherin switching [22]. These transcription factors also induce changes in the cytoskeleton that are necessary for the induction of motility [23], especially the expression of intermediary filaments like vimentin [24].

This review will first provide examples from the literature where developmental cues have inhibited aggressive melanoma progression. Then, to elucidate the nature of such cues, embryonic stem cell differentiation models towards melanocytes will be discussed. Furthermore, parallels between developmental stages of melanocyte differentiation and different tumor stages will be drawn. Finally, we will outline possible future melanoma treatment with regards to influencing melanoma differentiation and thereby inhibiting the dynamic process of phenotype switching.

## 2. Evidence for a Role of Development-Associated Factors during Melanoma Progression

During embryonic development there are numerous examples where the embryo microenvironment displays a tumor suppressive effect on melanoma cells. Early evidence for such a phenomenon was found when B16 murine melanoma cells were transplanted into the skin of mid gestation mouse embryos, which resulted in reduced tumor incidence [25]. Additionally, embryo-derived factors from the skin could also reduce tumor proliferation in vitro. When human melanoma cells were transplanted into chick embryos, the invading melanoma cells displayed neural crest-like characteristics and did not form tumors; instead, melanoma cells responded to cues from the surrounding host tissues and migrated along routes also used by neural crest cells [26]. In zebra fish, phenotype switching of melanoma cells was observed [27]. In this study extravasated and migration-arrested melanoma cells became differentiated and showed a highly proliferative status.

Considering the above examples of the microenvironment acting upon melanoma cells and altering their behavior, it is reasonable to conclude that specific developmentally controlled factors have the potential to influence human melanoma progression. Hence, understanding neural crest induction and further differentiation towards melanocytes could provide information on additional factors suitable for melanoma treatment. Additionally, model systems which enable the investigation of stem cell differentiation towards the melanocytic lineage are of great interest, since they allow the identification of such extrinsic differentiation factors under laboratory conditions.

## 3. In Vivo Differentiation of Melanocytes

**Figure 1:**
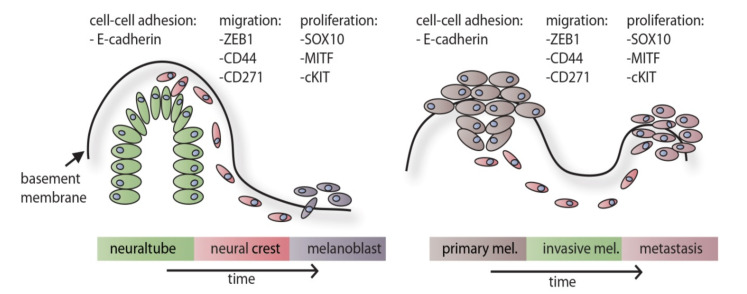
Comparison of embryonic melanocyte generation and the metastatic process in malignant melanoma. Left panel depicts neural tube formation and subsequent delamination. Migration of neural crest cells is indicated following terminal differentiation after invasion into the epidermis. Right panel depicts primary melanoma growth and subsequent migration and invasion of cells until melanoma cells colonize distant metastatic sites.

Melanocytes are cells of the neural crest, a unique ectodermal cell population of vertebrates derived from the outermost layer of the neural tube [28]. When the neural plate of the early embryo folds onto itself to become the neural tube, the small area of cells along the border becomes free from both the epidermis and the newly formed neural tube and undergoes EMT as described in Figure 1 on the left panel. These cells migrate through the body and differentiate into various cell types of the neural crest lineage: the cranial neural crest, the cardiac neural crest, the trunk neural crest, the vagal and the sacral neural crest [28]. The cranial neural crest differentiates further into cells of the thymus, some bones of the jaw and the odontoblasts, among others [29]. The cells of the cardiac neural crest form cartilage, connective tissue and differentiate into melanocytes of the head region [30]. The trunk neural crest forms cells of the adrenal gland, the nerves surrounding the aorta and also melanocytes (those of the rest of the body) [31]. The vagal and the sacral neural crest form the enteric ganglia [31,32].

Trunk neural crest cells are further divided into two distinct subpopulations—dorsal and ventral trunk neural crest—after the direction of their migration. The dorsal trunk neural crest differentiate into the main population of melanocytes, while the larger part of the ventral trunk neural crest produces neurons of the dorsal root ganglia, sympathetic chain and adrenal medulla, as well as a small subpopulation of melanocytes [33]. Melanocytes reach the skin during its innervation, as the Schwann cell precursors innervate the skin along with the cells of the ventral trunk neural crest that later become neurons [34]. This process of skin innervation, during which congenital or prenatal nevi appear [35], takes place in pregnancy weeks six to eight [34,35]. After the 13th week of pregnancy, the majority of melanocytes are located at their final destination [36].

Melanocytes are derived from glial-melanocyte precursors called melanoblasts, a stem cell population, which also gives rise to glial Schwann cells. A fully developed glia cell—if put into melanocytic medium—will give rise first to progenitor cells and later on to fully developed melanocytes [37]. Further evidence exists that melanocytic stem cells derived from the ventral trunk neural crest are also located in some cutaneous nerves and are regulated by nerve signaling [38].

Melanoblasts proliferation occurs within the same time period, not concomitantly but in an alternating cycle, as their migration into the skin. For this process to take place, multiple steps of changes in gene regulation need to occur. The master regulator of melanocyte development is the so-called microphtalmia-associated transcription factor (MITF), a basic helix-loop-helix leucine zipper transcription factor. Markers for melanocyte precursors include c-kit, SOX10, MITF, TYRP-2 and Pax 3 [39,40,41]. Migration is controlled by the extracellular matrix via receptors for a specific kind of endothelin (EDNRB2) and for ephrin (EphR) [42,43], although the process is yet to be fully understood. For the development of melanocytes from the neural crest, essential proteins including various endothelins, SCF (Stem Cell Factor), Wnt proteins and Neuregulin-1 [44,45] are needed; most of them will induce MITF expression [39,46]. This process is best described in other works, including [40,44,45,47]. The main marker for adult melanocytes is the enzyme tyrosinase, a protein which oxidizes tyrosine to become L-Dopa, the first step in the synthesis of melanin, the so-called melanogenesis, which is the melanocyte’s main task [48].

## 4. In Vitro 2D and 3D Differentiation Models for Melanocytes

Deriving melanocytes or melanoblasts *in vitro* from hESCs (human Embryonic Stem Cells) or hiPSCs (human induced Pluripotent Stem Cells) provides a valuable tool for studying melanocyte differentiation or to model pigmentation related diseases [49]. Differentiation protocols follow in general the same path as in vivo: hPSCs are first induced to differentiate into neural crest cells, then into melanoblasts and finally are matured into melanin producing melanocytes which correctly home to the basement membrane in an organotypic skin reconstruction assay [50,51].

The induction of neural crest cells at the neural plate border before undergoing EMT and migrating out of the neural tube relies on BMP, WNT, Notch/Delta and FGF signaling coming from the surrounding embryonic tissues [52]. Phenotypic differentiation into peripheral neurons, glia cells, bone and cartilage of the head, smooth muscle cells, melanocytes and endocrine cells will be strongly modulated by the neural crest cells spatial identity along the neural tube and onset of migration [53]. According to these developmental programs there are several ways of differentiating neural crest cells from hPSCs *in vitro*.

Early strategies relied on producing neural rosettes, neurospheres or embryoid bodies interspersed with neural crest cells by using dual-SMAD inhibition of BMP and Activin A/Nodal signaling and by promoting WNT signaling [53,54,55]. This was an inefficient and highly variable approach, as different groups used different markers for the isolation of neural crest cells (CD271, HNK-1 and SOX10 to name a few) and the role of the rosettes, neurospheres and embryoid bodies was not well defined in the induction. Another method is based on direct reprogramming of fibroblasts using transcription factor SOX10 and WNT activation to derive induced cells from human patients [56].

The preferred protocol for an adherent culture system today is based on the dual-SMAD-inhibition (DSi) conditions, known to play a role in the differentiation of hPSCs into the central nervous system lineages [57]. This protocol is optimized for melanocyte specification and has been used to model pigmentation defects in hESCs and patient-specific iPSCs [50]. In brief, hPSCs seeded on matrigel matrix are treated for 11 days with two small molecules (LDN193189 and SB431542) which inhibit BMP, TGF-b, activin and nodal signaling. This triggers the exit from the pluripotent state, prevents trophectoderm formation and blocks the formation of mesendoderm and non-neural ectoderm. A brief pulse of WNT activation afterwards will induce neural crest cells with high efficiency. This is achieved by CHIR99021, a WNT agonist, which selectively inhibits GSK-3b.

The dual-SMAD inhibition/WNT activation protocols derive a neural crest cell population biased towards the cranial (anterior) identity over the vagal (posterior) one. By adding retinoic acid and/or BMP to a neural crest induction protocol, vagal [58,59] and trunk [60] neural crest cells could be generated. While the generation of several neural crest derivatives is restricted to specific locations along the neural tube, melanogenic and neurogenic progenitors are produced along the entire axis [61], circumventing the need to specify regionally for the melanoblast precursors.

Melanocyte-competent subfractions of the induced neural crest cells are then isolated based on the presence of two transcription factors: c-KIT, which plays an important role in melanocyte migration, proliferation and maturation, and SOX10, which has major roles in the establishment and normal function of the melanocyte. Both these genes are known to be affected in melanoma patients [62,63]. After sorting, these cells are cultured in defined conditions using media that contains factors important for melanocyte maturation (SCF, EDN3, FGF2, CHIR, cyclic AMP, BMP4 and B27). First, pigmented cells, which contain melanophores appear on day 6 of melanocytic induction, with all the cells being pigmented by day 20. The gene expression profiles of these in vitro differentiated melanocytes showed that they are more similar to adult than neonatal primary melanocytes, although they lacked Hox genes expression [50].

Other protocols induce ectodermal differentiation and neural crest specification using human recombinant BMP4 and ascorbic acid, as well as Wnt3A, and make use of feeder cells for the seeding of hPSCs. This requires using FAD culturing media comprised of 2/3 DMEM, 1/3 HAM/F12, 10% FCS and other factors, such as insulin, hydrocortisone, triiodothyronine, adenine, recombinant human EGF and cholera toxin. Cells are grown until pigmented populations are observed, which are selected and amplified in melanocyte culture media M254 containing essential amino-acids, vitamins, inorganic salts, glucose, adenine, thymidine, sodium pyruvate, etc. This is supplemented with melanocyte growth factors: FGF2, insulin, transferrin, bovine, pituitary extract, DMSO, FBS, heparine, hydrocortisone and endothelin-1 [51,55].

A different and rather elaborate approach for generating melanocytes in vitro is represented by the organoid model. This co-induces both surface ectodermal and mesenchymal cells from hPSCs by using a stepwise modulation of the TGFb and FGF signaling pathways in a self-organizing system that mimics cell-to-cell signaling mechanisms needed for hair follicle induction. Cells are induced to become non-neural ectoderm with BMP4 and SB431542 (TGF-b inhibitor), upon which uniform epithelial cysts are generated. These are then treated with media containing FGF2 and LDN193189, a BMP inhibitor to induce cranial neural crest cells on the outer surface. An incubation period of 4–5 months in maturation media and constant shaking yields inside-out cyst-like skin organoids consisting of stratified epidermis, fat-rich dermis and pigmented hair follicles equipped with sebaceous glands. These organoids could be integrated in the endogenous skin of a mouse model and grew hairs a month after the transplantation in 55% of the cases [64].

## 5. Parallels between Neural Crest Development and Melanoma Progression

The EMT phenotype reflects a cellular status which confers invasive abilities. Additionally, it has been found to be linked with the cell cycle. In embryo development it seems that for the initiation of migration, entry into cell cycle is of advantage, but during the migrative process, the majority of cells do not cycle. It has been shown that trunk neural crest cells, when they delaminate from the dorsal portion of the neural tube to migrate, do so in the S phase of the cell cycle [65], while cranial neural crest cells do not depend on the cell cycle for delamination and migration [66]. Another study using the FUCCI reporter showed that most cranial neural crest cells are quiescent during migration, and that when the destination was reached, some cells showed rapid proliferation, while others exited the cell cycle [67]. Attempts have been made to identify gene expression signatures of trunk neural crest cells after they settled at selected sites and initiated differentiation [68].

In cancer, EMT has often been found associated with reduced cell cycle [69,70]. Specifically in melanoma, the transcription factor MITF is impacted by the EMT process, with ZEB1 and HIF1A being potent repressors of MITF expression [71,72]. A schematic, including exemplary factors, during embryonic development and melanoma progression is shown in Figure 1.

Importantly, the presence of MITF, as well as SOX10, is crucial for lineage commitment and cell proliferation [73]. SOX10 binds in the promoter region of MITF and hence is expressed in all congenital naevi and melanomas. During development, it regulates the balance between neural stem cells maintenance and multi-lineage differentiation and has a key role in the formation of pigmented cells. In melanoma, SOX10 is essential for the maintenance of proliferation. In vitro studies have shown that silencing this transcription factor results in suppression of neural stem cell properties, cell proliferation and survival, completely abolishing in vivo tumor formation [74].

Melanoma cells are characterized by an either high or low MITF expression signature, which confers the following corresponding functional characteristics: strong MITF expressing cancer cells are highly proliferative, but neither invasive nor metastatic. On the other hand, low MITF expressing cells frequently give rise to metastatic lesions, while the cell cycle is strongly reduced in this tumor cell sub-population [75,76]. In addition to its effect on proliferation, melanoma cells low in MITF also show strong resistance to multiple targeted drugs against melanoma [77].

It is important to note that MITF expression can also be induced in progenitor cells with the help of BMP4; however, it needs the help of cofactors induced by distinct signaling pathways like endothelin 3 to promote the differentiation of precursors into mature melanocytes [78].

An important characteristic of cells that undergo EMT and disseminate, is the expression of stem cell markers previously encountered during development, which are associated with stemness and tumorigenicity. This aberrant re-activation of the embryonic neural crest program alters the cell metabolism as well as behavior, lifting the paracrine control of the surrounding keratinocytes, while acquiring autocrine signaling [79,80]. Stem cell markers present in melanoma cells are nestin, CD133, CD271 and Sox2.

Nestin, a neuroectodermal stem cell marker, is an intermediate filament protein expressed in the cytoplasm of neuro-epithelial stem cells and the endothelium of growing blood vessels. It cannot be detected in differentiated neuronal cells and normal melanocytes, but re-appears in benign and malignant melanocytic tumors and usually predicts poor prognosis in human melanoma [81].

CD133, also called prominin-1, is a transmembrane glycoprotein expressed on hematopoietic stem cells, endothelial progenitor cells and dermal derived stem cells capable of differentiating into neural cells. It is considered one of the most important cancer stem cell-associated marker identifiers so far, being present in a wide variety of solid tumors (brain, prostate, pancreas, glioblastoma, melanoma and gut) [82,83]. Upon Wnt signaling, it influences cell polarity, migration and the interaction of cancer stem cells with both surrounding cells and ECM (Extra Cellular Matrix), highly increasing their metastatic potential [84,85].

CD271 (NGFR/p75NTR) acts as a molecular switch with divergent roles in melanocyte development and melanoma. During embryonic development, neural crest cells and melanoblasts express the growth factor receptor CD271 responding to the action of neutrophins through several canonical pathways, including MAPK and PI3K/AKT. For the melanocytic lineage this mediates cell growth, survival and differentiation. Melanoma cells that show high levels of CD271 are associated with metastatic progression, enhanced survival, resistance to chemotherapeutic agents (MAPK inhibitors) and evasion of the immune system [86,87].

Sox2 is an embryonic stem cell marker essential for the self-renewal of embryonic stem cells [88]. During embryogenesis, it is expressed throughout developing cells of the neural tube, as well as in the progenitors of the nervous system, until cells differentiate and become post mitotic [89]. Sox2 is highly expressed in melanoma stem cells contributing to dermal invasion and primary tumor thickness [90]. Mechanistically, Sox2 binds to an enhancer region of the gene that codes for nestin and positively influences its expression. In human melanomas, the co-expression of these two factors serves as an indicator of the tumor’s aggressiveness [91]. Importantly, SOX2 expression in melanoma is up-regulated by STAT3 activity [92].

Another key player in the developmental and malignant processes is the extra-cellular matrix (ECM). Under normal conditions, surrounding keratinocytes control the growth rate and phenotype of melanocytes in the epidermis in an E-cadherin dependent fashion. They secrete endothelins, short peptides which stimulate proliferation, chemotaxis and pigment production and survival in melanocytes through the Endothelin Receptor Type B (ET_B_). During melanomagenesis, E-cadherin, a tumor invasion suppressor, is down-regulated, epigenetically and environmentally, and melanoma cells become refractory to the keratinocyte control. Activation of ET_B_ pathway by ET1 and ET3 contributes to the down-regulation of mRNA and protein levels of E-cadherin, as well as of α- and β-catenin adhesion proteins [93].

BMP4/7 stimulates the expression of Id (inhibitor of differentiation/DNA binding) proteins through its interaction with a number of ECM components, particularly hyaluronan (HA), present in high amounts in the skin. Increased levels of BMP4,7/Id axis contributes to malignancy in melanoma by promoting stemness and tumor initiation [94]. HA is a linear high molecular weight polysaccharide synthesized by three transmembrane synthases HAS 1,2,3 and is extruded into the ECM. Melanoma cells synthesize large amounts of HA in early tumorigenesis and with tumor-associated fibroblasts continuing to produce this molecule later on. HA promotes BMP4/7-dependent Id1/3 protein expression in melanoma cells by binding its cellular receptor CD44 and directly aids in cell proliferation, motility, invasion and metastasis. Loss of HA from the ECM can reduce Id1/3 protein expression.

Last, but not least, hypoxia, among micro-environmental/ECM components, represents a key-tumor promoting factor. Mild hypoxia is essential for melanocyte transformation, as only under hypoxic conditions does PI3K-Akt stabilize and up-regulate HIF-1α [95]. This transcription factor is key in the progression of melanoma, as it allows adaptation to hypoxia, angiogenesis, glucose utilization and switching on of survival factors, especially of the transferrin receptor, as cancer cells have a much higher metabolic iron requirement [96,97].

Taken together, the physiologic processes of neural crest delamination show many similarities to those found in melanoma cells in the EMT status. It is plausible that the cell cycle is down-regulated during the migration phase and activated again when the destination site is reached. Accordingly, MITF, the master regulator, is expressed in developing melanocytes similarly to its expression in highly proliferative melanoma cells. However, aggressive melanoma is hallmarked by its ability to repeat this phenotype switching process multiple times by using both paracrine and autocrine signals, as well as hijacking signaling pathways active in stem and progenitor cell populations during normal development. The molecular switch which triggers the EMT process has ultimately not been identified, but candidate transcription factors as well as receptors and ligands are shown in Table 1. 

## 6. A Working Model for Melanoma Progression

Melanomagenesis can be seen as a de-differentiation process of mature melanocytes, where altered melanocyte stem cells or immature progenitors hijack signaling pathways active in stem or progenitor cell populations [81]. This process can more readily occur in melanocytes, since as a result of their developmental program, they harbor a high degree of plasticity and invasiveness. Hence, when under appropriate conditions, melanoma cells can re-activate developmental programs in order to further progress in malignancy [79].

The initial niche of melanocytes is within keratinocytes in the stratum basale at the bottom of the epidermis. Keratinocytes supply melanocytes with an array of signalling molecules that regulate their proliferation and differentiation in a paracrine manner. Additionally, UV radiation triggers the release of pro-, but also anti-inflammatory, cytokines. The ligands secreted by keratinocytes include SCF, basic FGF (FGF2), keratinocyte FGF (FGF7), interleukines (such as IL6 and IL18), endothelines (ET1 and ET3) and bone morphogenic proteins (BMP4 and BMP7). These factors are generally secreted in response to UV radiation and melanocytes are under paracrine control by expression of the corresponding receptors: c-kit for SCF, FGF receptor, endothelin receptor and others.

According to the cancer stem cell theory, there are two possible tumor initiators within the melanocytic niche. One is represented by the melanoblasts or immature progenitors, comprising cells with a low differentiation level, but high self-renewal potential *in vivo* and high tumorigenic potential, while the others are the melanocytes themselves, a more differentiated cell population with only low potential of renewal. In general, melanoma initiating stem cells are positive for stem cell markers, such as CD271 and CD133 and exhibit morphological, phenotypic and functional features of a stem cell population. Cells positive for these markers are capable of generating secondary tumors in nude mice [107].

The microenvironment of melanoma-initiating cells contains, besides keratinocytes, also fibroblasts, endothelial cells and immune system cells, which provide a rich repertoire of secreted molecules which aid in cell motility, angiogenesis and invasion [80]. The cancer cells themselves secrete soluble factors to prepare their homing site even before they reach it, such as VEGF, GCSF, FGF2, PDGF and TGF-β [146]. These factors, and others, alter the ECM and recruit myeloid cells with immune-suppressive properties, so-called myelo-derived suppressive cells, tumor-associated macrophages or tolerogenic dendritic cells. This process enables the formation of metastases and protects tumor cells from the immune system [147]. Immune privileged sites, such as the eye and the brain, seem to be preferred colonization sites by melanoma cells. Metastatic spread is considered to be the most inefficient step in melanoma progression [148]. Still metastasis is the major reason why patients succumb to this often fatal disease. Inhibition of the metastatic process is the major aim for the future and insights into developmental cues may hold the key for novel and effective therapeutic approaches.

## 7. Conclusion and Possible Therapeutic Options for Future Melanoma Treatment

**Figure 2:**
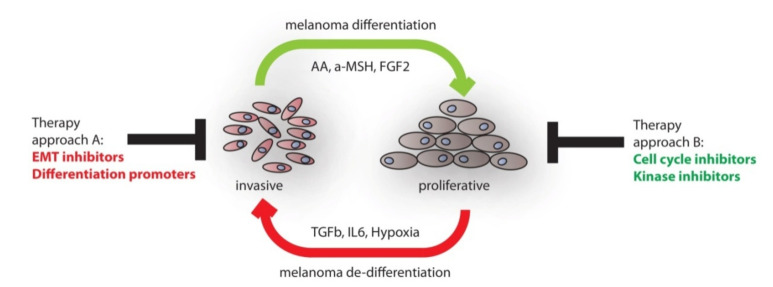
Dynamic process of phenotype switching in human melanoma. Proliferative melanoma cells become invasive and migrative upon stimulation with extrinsic factors. In turn, invasive melanoma cells switch to a more differentiated and proliferative phenotype when treated with ascorbic acid (AA), the hormone a-MSH or the growth factor FGF-2. Therapeutic options include (**A**) treatment of invasive cells with epithelial mesenchymal transition (EMT) inhibitors or differentiation promoters and (**B**) treatment of proliferative cells with cell cycle or kinase inhibitors. Importantly, therapy should stop the continuous switching between cellular states. AA: Ascorbic Acid, a-MSH: α-Melanocyte stimulating hormone, FGF2: basic fibroblast growth factor, TGFb: transforming growth factor beta, IL6: interleukine 6.

Here, we reviewed, on the one hand, the developmental process of neural crest induction and, on the other hand, discussed factors which contribute to melanocytic differentiation. We have summarized molecular clues instrumental for establishing neural crest and melanocyte progenitor cells. A number of molecular markers are available to identify these cell populations as already outlined in Table 1. The transition from the progenitor pool to differentiated melanocytes is accompanied by up-regulation of the MITF pathway, which controls pigmentation, but also other melanocyte specific characteristics. The knowledge gathered from developmental programs occurring during embryonic skin development can be used in order to gain mechanistic insights into the process of malignant melanoma formation and progression. Especially, the metastatic process in melanoma can be associated with a switch in developmental states. On the one hand, melanoma cells in the migrative and invasive phase express prominent neural crest cell marker profiles, including genes controlling stemness. On the other hand, cells which stopped migrating and adopted a proliferative phenotype express differentiation associated genes. A model for the dynamic process of phenotype switching is shown in Figure 2.

Given the evidence presented that melanoma cells start re-expressing neural crest stem cell markers and behave according to an embryonic development program, the question remains whether differentiation of melanoma cells into melanocyte-like cells would represent a valuable therapeutic strategy. Since forced differentiation of melanoma cells is a difficult task, researchers have manipulated individual factors and thereby influenced melanoma outcome. In the following, we present several treatment approaches targeting melanoma stemness or differentiation factors.

MITF is the master regulator for the melanocyte lineage. Its down-regulation by siRNA in murine melanoma cells lead to the re-expression of pluripotency markers OCT4 and NANOG, together with an increased invasive phenotype [149]. A similar effect is generated by treatment of melanoma cells with TNF-alpha, which mimics an immunotherapy induced inflammation. There, a de-differentiated phenotype was induced with down-regulation of pigmentation-related genes and up-regulation of the stemness marker CD271 [150]. CD44 is also designated as a stemness marker and its regulation influences melanoma fate. As an example, forced expression of a secreted and mutated form of CD44 completely abolished melanoma cell adhesion to immobilized hyaluronic acid and inhibited the hyaluronic-acid induced proliferation of melanoma cells *in vitro* and *in vivo* [104]. Interestingly, down-regulation of another stemness marker CD-133 via shRNA resulted in a reduced capacity of the cells to metastasize and decreased ability to form spheroids under stem cell-like growth conditions of a human melanoma cell line [84].

α-Melanocyte stimulating hormone (α-MSH) is secreted by normal human keratinocytes and melanocytes and has pigmentary, anti-inflammatory, antipyretic and immunoregulatory roles. This peptide also acts as a paracrine regulator of proliferation and differentiation for both melanocytes and melanoma cells and in early melanoma stages it inhibits tumor evasion [102]. Importantly, α-MSH stimulates the melanocortin-1 receptor and triggers increased pigmentation and control of senescence. Accordingly, it has been shown that melanocortin-1 activity prevented melanomagenesis in mice [151]. In the past, the presence of pigmentation and in particular expression of MART-1 (MLANA)-derived peptides has been used to generate vaccines against melanoma (e.g., NCT00006385, NCT00923195). However, significant clinical benefit was not achieved [152].

Interference with FGF2 signaling has so far not been reported to change overall melanoma differentiation. However, blockade of FGF signaling resulted in reduced proliferation and increased cellular apoptosis [136]. Additionally, a FGF2-derived peptide that inhibits FGF2 dimerization and acts as a molecular decoy has inhibited melanoma growth *in vitro* and *in vivo* [137].

Another potent substance is sodium ascorbate, because it showed promising results in inhibiting the invasion and growth of melanoma cells. Tumor cell responsiveness is based on multiple effects, including alteration of cellular reactive oxygen species amounts and inducing cellular apoptosis. For example, it was shown that ascorbic acid decreases cellular iron uptake in a dose- and time-dependent fashion by decreasing the transferrin and melano-transferrin surface receptors [111]. Iron is a rate-limiting nutrient for growth and the expression of an extra transferrin receptor may provide the neoplasic cells with a competitive advantage against normal cells, which could account for their highly aggressive nature *in vivo* [110]. Sodium ascorbate induced apoptosis can be enhanced by the iron chelator desferrioxiamine and inhibited by the iron donor ferric ammonium citrate. Moreover, ascorbic acid serves as an essential co-factor for prolylhydroxylase (PHD) enzymes, which post-translationally target HIF-1α for degradation [153]. Using ascorbic acid in a mixture containing lysine, proline, arginine and green tea extract inhibited pulmonary metastasis of melanoma B16FO cells in C57BL/6 mice. This could additionally be explained through the roles lysine and ascorbic acid have in maintaining the stability of connective tissue as natural inhibitors of ECM degradation; the synthesis and structure of collagen fibrils depends upon hydroxylation of proline and lysine residues by ascorbic acid [154].

Melanoma de-differentiation can be triggered by inflammatory and cellular stress signals like transforming growth factor beta (TGF-beta), hypoxia and Interleukin-6 (IL-6).

TGF-beta signaling induces transcription factors like SNAIL, SLUG and ZEB1, which repress E-cadherin. Importantly, TGF-beta activity has been shown to reduce MITF amounts and to induce an invasion process [155]. While repression of TGF-beta signaling decreased invasion [156]. Hence TGF-beta is considered a main driver of the EMT process. Patients suffering from advanced malignant melanoma were treated with a TGF-beta neutralizing antibody (NCT00356460). Preliminary evidence of antitumor activity was observed and further combination treatments are expected [157]. Recent work has shown that along with TGF-beta the hypoxia induced transcription factors (HIF) are also activated [158]. HIFs are very important for the metastatic process and as indicated above oxygen as well as reactive oxygen species regulate PHD activity, which determines stability of HIF proteins.

Interleukin-6 (IL-6) is a cytokine strongly expressed in human malignant melanoma cell lines [117]. IL-6 acts as a growth inhibitor for melanoma cells in early stages, but IL-6 can stimulate cells derived from advanced lesions. Hence, addition of purified recombinant human IL-6 resulted in a growth inhibition *in vitro* by G1/G0 arrest of early, but not advanced stage melanomas [115,118]. Additionally, STAT3 activation by IL6 was shown to reduce MITF levels, but enhance NANOG and OCT4 levels, which was reminiscent of a tumor-initiation phenotype [135]. A study on the interleukin-6 receptor inhibitor Tocilizumab in combination with Ipilimumab and Nivolumab in patients with stage III or stage IV melanoma is in the phase of recruiting (NCT03999749).

Currently, melanoma patients frequently suffer from acquired resistance towards systemic targeted therapy [159]. In melanoma combined treatment with BRAF and MEK inhibitors only extended median progression-free survival, from 7.3 months, in the monotherapy group, to 11.4 months in the combination therapy group [160]. Resistance is established by reactivation of RAF-MEK-ERK signaling and, besides mutational changes, the acquisition of an EMT phenotype has been shown to enable this process [161,162]. Hence, there is an urgent need to improve the current situation. As depicted in Figure 2, molecular targets are available which provide multiple opportunities for therapeutic options. This can be achieved by interfering with the phenotype switching process in melanoma and thereby blocking the continuous cycling between cellular states. A synergistic approach should be considered, where cells are blocked to reach the invasive, neural crest like status. Thereby, cells would return to a differentiated and possibly highly proliferative phenotype. Targeted therapy should be highly effective in continuous proliferating cells, since the generation of dormant, EMT-like stem cells could be largely excluded [163]. Ways to achieve changes in cellular differentiation include the use of neutralizing antibodies directed against extracellular ligands or pharmacologic inhibition of signaling pathways. Endogenous proteins, such as transcription factors, are difficult to target. However, recently, the field of targeted proteolysis has evolved into a promising technology for novel therapeutic applications [164]. Importantly, the method using proteolysis-targeting chimeras (PROTACs) does not require prior genetic modification of target proteins. Instead, small molecules are used, which specifically bind endogenous target proteins as well as an E3ligase component. This leads to ubiquitination and degradation of the target proteins. A PROTAC class of molecule was used to degrade the transcription factor BRD4 in Vemurafenib resistant melanoma cells, which leads to severely reduced colonogenic growth activity [165].

Finally, understanding the dynamics of melanocyte differentiation holds great promise for revealing further insights into mechanisms of melanoma progression. The identification and targeting of key factors, driving phenotype switching during melanoma development, might lead to more effective strategies in combating this fatal disease.

## Figures and Tables

**Table 1:** List of molecules contributing either to a differentiated, proliferative phenotype or to a migrative, neural crest phenotype.

Category	Name	Proliferative, Differentiated	Migrative, Progenitor	References
TF	SOX10	+ +	+	[74,98]
TF	SOX2	+	+ +	[90,91,99]
TF	MITF	+ + +	+	[73,100,101]
hormone	a-MSH/Melanocortin	+ + +	+	[80,102]
receptor/ligand	CD44/Hyaluronic acid	+	+ +	[94,103,104,105,106]
receptor	CD271	+	+ + +	[86,87,107]
receptor	CD133	+	+ +	[84,108,109]
co-enzyme	Iron	+ +	+	[110,111]
TF	HIF-1α	+	+ +	[96,97,112]
ligand	BMP-4	+ +	+ +	[78,113,114]
cytokine	IL-6	+	+ + +	[115,116,117,118]
filament	Nestin	+	+ +	[81,119]
hormone	IGF-1	+	+	[120,121]
ligand	Endothelin 1, 3	+	+ +	[122,123]
receptor	Endothelin receptor	+ +	+	[124,125]
receptor	c-kit	+ +	+	[126,127,128,129]
ligand	SCF	+ +	+	[130,131,132]
TF	STAT3	+	+ +	[133,134,135]
ligand	FGF2	+ + +	+	[136,137,138,139,140]
ligand	FGF7	+ +	+	[141,142,143]
TF	Id1, Id3	+	+ +	[94,144,145]

TF: transcription factor.
